# Supplementary figures and images for: Neonatal subarachnoid hemorrhage disrupts multiple aspects of cerebellar development
Source: Front Mol Neurosci. 2023 Apr 28;16:1161086. doi: 10.3389/fnmol.2023.1161086 (PMC10175619; doi:10.3389/fnmol.2023.1161086)

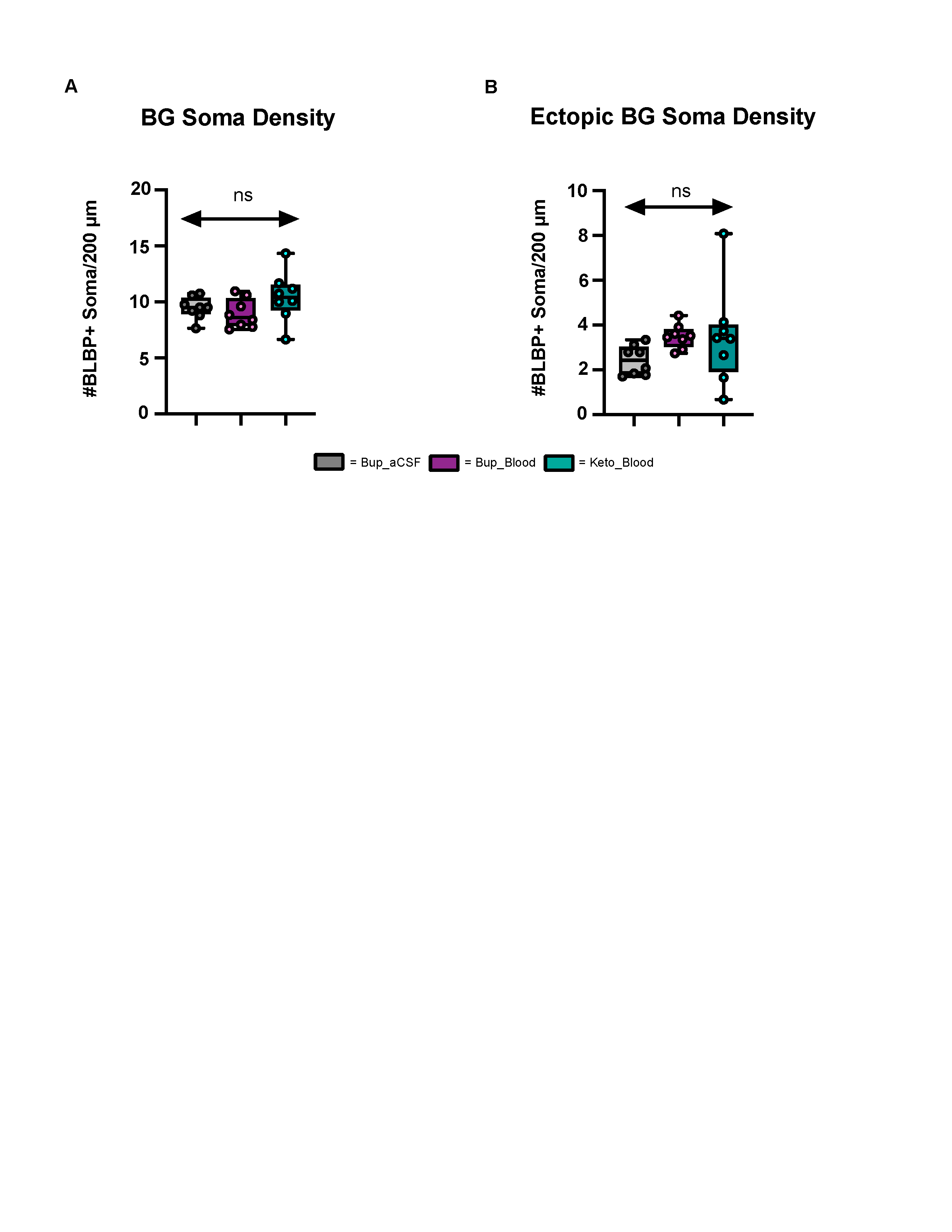

Supplement: Supplementary Figure 1 — CHI did not result in significant differences in BG soma density or ectopic positioning of BG soma at P42. (A) Quantification of BG soma density for all treatment groups (N = 1, n = 9). (B) Quantification of BG soma density noted in ectopic positioning for all treatment groups (N = 1, n = 9). Significance defined as p < 0.05 by one-way ANOVA with Tukey's post-test. Data are presented as SEM with minimum and maximum values. N = number of animals per group included for analysis, n = number of sections per group included for analysis. [file Image_1.TIFF]
